# Synthesis and characterization of Se doped Fe_3_O_4_ nanoparticles for catalytic and biological properties

**DOI:** 10.1038/s41598-023-28284-x

**Published:** 2023-01-18

**Authors:** Mohammad Reza Ahghari, Zeinab Amiri-khamakani, Ali Maleki

**Affiliations:** Catalysts and Organic Synthesis Research Laboratory, Department of Chemistry, Iran University of Science and Technology, Tehran, 16846-13114 Iran

**Keywords:** Chemistry, Nanoscience and technology

## Abstract

In this study, Se-doped Fe_3_O_4_ with antibacterial properties was synthesized using by a coprecipitation method. The chemistry and morphology of the Se doped Fe_3_O_4_ nanocomposite were characterized by energy-dispersive X-ray spectroscopy, field-emission scanning electron microscopy, X-ray diffraction, vibrating sample magnetometry, and Brunauer–Emmett–Teller spectroscopy. The antibacterial activity of the Fe_3_O_4_/Se nanocomposite was examined against G^+^ (Gram-positive) and G^−^ (Gram-negative) bacteria, in the order *Staphylococcus* *aureus*, *Staphylococcus* *saprophyticus*, *Pseudomonas* *aeruginosa*, *Klebsiella* *pneumonia*, and *Escherichia* *coli*, which are the most harmful and dangerous bacteria. Fe_3_O_4_/Se, as a heterogeneous catalyst, was successfully applied to the synthesis of pyrazolopyridine and its derivatives via a one-pot four-component reaction of ethyl acetoacetate, hydrazine hydrate, ammonium acetate, and various aromatic aldehydes. Fe_3_O_4_/Se was easily separated from the bacteria-containing solution using a magnet. Its admissible magnetic properties, crystalline structure, antibacterial activity, mild reaction conditions, and green synthesis are specific features that have led to the recommendation of the use of Fe_3_O_4_/Se in the water treatment field and medical applications. Direct Se doping of Fe_3_O_4_ was successfully realized without additional complicated procedures.

## Introduction

In recent years, nanotechnology has been hailed as a ground-breaking field that ushered in a new era in applied sciences and technology^1,2^. Synthesized nanomaterials have many broad applications in a variety of fields, including pharmaceutical, medical, cosmetic, and electrical applications. Of course, the materials are classified as nanoscale with at least one dimension of less than 100 nm. These materials were considered when researchers discovered that the size had a significant impact on the physical and chemical properties of the material^3,4^. Nanomaterials have a lot of fantastic and unique properties including that some of these materials have the potential ability to eliminate bacterial infections. Since today in the world because of excessive antibiotics, the growth of infectious bacteria resistant to these drugs is increasing day by day, the ineffective of these drugs against infectious bacteria is a serious threat to the world. However, some nano-sized compounds have appeared against such very strong bacteria, which has led to the attention of the global scope to nanomaterials^5–10^. One of the compounds that has long been known to humans is magnetite, Fe_3_O_4_, which is a common magnetic iron oxide with an inverted cubic spinel structure created by oxygen-iron bonding, where Fe (III) ions occupy both tetrahedral and octahedral sites. Fe_3_O_4_ nanoparticles are one of the most widely used magnetic compounds owing to their various applications and remarkable properties^11–14^. Functionalization of the surface of iron oxide nanoparticles with biocompatible materials increases the properties of this material, including colloidal stability and reduction of cytotoxicity^15,16^. Selenium is one of the most essential trace elements, in the human body and is an enzymatic antioxidant^17–19^; particularly vulnerable patients suffer from phenylketonuria, so individuals with diet-related diseases experience severe selenium deficiency. Additionally, individuals exposed to specialized chemotherapy and those who have already undergone radiotherapy are vulnerable to a decrease the amount of this element in vulnerable organisms. Selenium has biological activities, including antibacterial, anti-inflammatory, antiviral, antiparasitic, antifungal, and anticancer activities^20–22^. In addition this element is nanosized, due to its proper interaction with functional groups (C–O, C–N., NH, and COO–) of proteins that possess good adsorptive and biological activity^23–26^. The application of selenium nanoparticles in the field of biomedicine because of their unique properties, such as nutritional properties, environmental friendliness, and good chemotherapy, is very noticeable^27–29^. Selenium, because of its antioxidant properties, increases cellular defense against oxidative stress in the cells of living organisms, including humans^30–33^. The mechanism of action of selenium is as follows: selenium functions as a redox center^24,34^; the best-known example of this redox function is the reduction of hydrogen peroxide and damaging lipid and phospholipid hydroperoxides to harmless products (water and alcohols) via the family of selenium-dependent glutathione peroxidases^2,24,35,36^. This function helps maintain membrane integrity, protects prostacyclin production, and reduces the likelihood of propagation of further oxidative damage to biomolecules such as lipids, lipoproteins, and DNA with the associated increased risk of bacterial infections, atherosclerosis, and cancer^23,36^. Drinking water is one of the most important and consumed substances by living organisms, and the life of most living organisms depends on water^37–40^. Drinking water is a suitable environment for growing bacteria, due to various factors such as inadequate disinfection^41–43^, the proper temperature, and the presence of nutrients, which causes water pollution and is a danger to human health^44–46^. Some hazardous bacteria found in water include G^+^ *Staphylococcus* *saprophyticus*, *Staphylococcus* *aureus*, and G^−^ *E. coli,* bacteria. In addition to water contamination, these bacteria are among the main causes of food poisoning^47–51^. Common Gram-negative bacteria. *Pseudomonas aeruginosa* is an opportunistic infection that is a major cause of illness and death in patients with cystic fibrosis and immunocompromised patients. The eradication of *P. aeruginosa* has become increasingly challenging because of its extraordinary ability to withstand medicines^52,53^.

*Pseudomonas* *aeruginosa* strains have a high degree of innate and acquired resistance mechanisms, which they use to evade most drugs^54^.

Furthermore, among Gram-negative bacteria, *Klebsiella pneumonia* is the predominant ESBL producer associated with urinary tract infection, and sometimes it progresses to more important infections, such as blood poisoning^55,56^.

Therefore, the G^−^ *E. coli* bacterium is the most common cause of urinary tract infections^57^, and the G^+^ *S. aureus* bacterium that causes skin infections such as abscesses, respiratory infections, including sinusitis, and food poisoning. Infections are frequently facilitated by pathogenic strains that release virulence factors, such as strong protein toxins, and produce a cell surface protein that binds and inactivates antibodies^58^. However, owing to their strong antibacterial properties, selenium-containing compounds can help remove bacteria from water. Selenium-containing compounds play an important role in organic synthesis, biochemistry, medicinal chemistry, and materials science. They can be used as nucleophiles, electrophiles, ligands, and catalysts in organic syntheses^59^. Selenium-catalyzed reactions have attracted extensive attention because of the advantages of mild and eco-friendly reaction conditions, good functional compatibility, excellent selectivity, high atom economy, and low cost. In the synthesis of biologically active organic compounds, multicomponent reactions (MCRs) with attributes such as experimental simplicity, synthetic efficiency, and formation of several bonds in one unit operation have been suggested, particularly in the case of heterocyclic compounds, such as pyrazolopyridine derivatives which are an essential system because of their confirmed utility as organic fluorophores, bioactive compounds, and ligands to coordination complexes^14,60,61^. These derivatives are an important class of nitrogen-containing heterocyclic chemicals with antileishmanial, antibacterial, and antiviral properties. Because the pyrazolopyridine nucleus is structurally similar to purines, its derivatives may compete with purines and prevent nucleic acid production. Several pyrazolopyridines have been to interact with DNA and limit the proliferation of cancer cell types^62–65^. By combining materials with potential properties, new materials with better and more effective performance can be created. In this study, the surfaces of Fe_3_O_4_ nanoparticles were coated with selenium nanoparticles, as a result of the performance of the new material synthesized as a nanocomposite in two different departments. The removal of bacterial contaminants from drinking water can also, be easily isolated from solutions containing bacteria using a magnet. The nanocomposite effectively killed pathogenic human bacteria. Additionally, it is a powerful catalyst for the synthesis of organic compounds with drug properties.

## Experimental

### General

Chemical materials were produced from Sigma–Aldrich, and Merck were purchased and used without additional purification. The following is a list of analytical device information: The TESCAN4992 instrument was used to record the energy-dispersive X-ray (EDX) analyses. Using a TESCAN4992, powder X-ray diffraction (XRD) patterns of the nanocomposites. The specific surface area and degree of porosity of the prepared samples were determined using the Brunauer–Emmett–Teller adsorption–desorption isotherm (BET., Micrometics ASAP2020). A Sigma-Zeiss microscope was used to capture pictures of the nanocomposite using field emission scanning electron micrographs (FESEM). An accurate magnetometer (Iran Kavir VSMs) was used to measure the magnetic properties of the solid object. The melting points were calculated using an electrothermal 9100 device. ^1^H and ^13^C nuclear magnetic resonance (NMR) spectra were recorded on a Bruker DRX-300 Avance spectrometer at 300 MHz and 75 MHz, respectively. The antibacterial activity of the nanocomposite against the pathogenic bacteria *Staphylococcus aureus *(*S. aureus*) (ATCC 12,600), *Staphylococcus saprophyticus *(*S. saprophyticus*) (ATCC 1440), G^−^ *Escherichia coli *(*E. coli*) (ATCC 9637*)*, *Klebsiella pneumonia *(*K. pneumonia*) (ATCC 700,603), and *Pseudomonas aeruginosa *(*P. aeruginosa*) (ATCC 27,853) was studied.

### Syntheses of Fe_3_O_4_/Se

First, using the sedimentation method, distilled water (200 mL) was used to dissolve the co-deposition of FeCl_3_·6H_2_O (20 mmol) and FeCl_2_·4H_2_O (20 mmol) salts, and the mixture was stirred at room temperature for approximately 50 min under a N_2_ atmosphere. The temperature was gradually increased to 85 °C. Subsequently, 10 mL of ammonium hydroxide (25%) was added dropwise to the stirring solution to provide iron oxide NPs at pH = 12. Finally, the Fe_3_O_4_ Nps magnetic dark precipitate was collected by an external magnetic field and washed with deionized water three times. In the continuous synthesis step, Fe_3_O_4_ NPs were dispersed in 10 ml of ethanol under intense stirring, and then, 1 g of selenium dioxide was added to the mixture and stirred at room temperature for 30 min. Sodium borohydride (0.03 g) was added and the mixture was stirred at room temperature. The prepared product was collected using an external magnetic field, washed with water and ethanol several times, and dried to afford a brown solid.

### Common procedure for the synthesis of pyrazolopyridine derivatives 5a–j

The catalytic activity of the Fe_3_O_4_/Se nanocomposite was tested in a one-pot synthesis of pyrazolopyridine derivatives. The four components were mixed and reacted in the presence of 1 ml EtOH at room temperature: hydrazine hydrate (2 mmol), ethyl acetoacetate (2 mmol), aromatic aldehydes (1 mmol), and ammonium acetate (3 mmol) (Table1). Thin-layer chromatography was used to evaluate reaction completion process (TLC). The undissolved magnetic nanocatalyst was separated from the reaction mixture using a magnet after completion of the reaction. To obtain pure pyrazolopyridine derivatives, the crude product was recrystallized from EtOH. All the products were well-known chemicals recognized by comparing their melting points with those in the original literature (Table 3).

**Table 1:** Surface area, pore volume, and pore diameter of the Fe_3_O_4_/Se nanocomposite.

Sample	Surface area^a^ (m^2^/g)	Pore volume^b^ (cm^3^/g)	Pore size^b^ (nm)
Selenium-coated Fe_3_O_4_	11.57	0.073406	6.95

^a^The surface area was obtained by BET analysis.

^b^Pore volume and pore diameter obtained by BJH analysis.

#### Spectral data of selected products

##### Spectral data of selected products. Analysis of the results of NMR

3, 5-Dimethyl-4-phenyl-1, 4, 7, 8-tetrahydrodipyrazolo [3, 4-b: 4′, 3′-e]Pyridine (5a): 1H NMR (300.13 MHz, DMSO-d6): δ = 2.06 (s,6H, CH3), 4.81 (s, 1H, CH), 7.14–7.20 (m, 5H, H-Ar), 11.24–11.32 (br s, 3H, NH); 13C NMR (75.47 MHz, DMSO-d6): δ = 10.8, 33.1, 104.6, 125.8, 127.9, 128.1, 140.2, 143.7, 161.5. IR (KBr: ῡ /cm − 1): 3528, 3301, 2925, 1614, 1515 and 1375.

4-(2,4-Dichloro-phenyl)-3, 5-Dimethyl-1, 4, 7, 8-tetrahydrodipyrazolo [3, 4-b: 4′, 3′-e]pyridine(5b):1H NMR (300.13 MHz, DMSOd6): δ = 1.95 (s, 6H, CH3), 5.06 (s, 1H, CH), 7.33 (dd, J1 = 2.1 Hz,J2 = 8.4 Hz, 1H, H-Ar), 7.47 (d, J = 2.1 Hz, 1H, H-Ar), 7.49 (d ,J = 8.4 Hz, 1H, H-Ar), 11.08 (br s, 3H, NH); 13C NMR(75.47 MHz, DMSO-d6): δ = 15.62, 36.37, 107.01, 131.71,133.56, 136.19, 137.10, 138.41, 143.80, 145.18,165.75.

4-(2-nitro-phenyl)- 3, 5-dimethyl-1, 4, 7, 8-tetrahydro-dipyrazolo [3, 4-b: 4′, 3′-e] pyridine(5c): 1H NMR (300.13 MHz, DMSO-d6): δ = 1.92 (s, 6H, CH3), 5.44 (s, 1H, CH), 7.37–7.68 (m, 4H, H-Ar), 10.98 (br s, 3H, NH); 13C NMR (75.47 MHz, DMSO-d6): δ = 10.0, 28.9, 101.9, 123.8, 127.1, 130.2,131.6, 136.2, 138.6, 149.5, 160.5.

4-(4-Bromo-phenyl)-3,5-dimethyl-1,4,7,8-tetrahydro-dipyrazolo[3,4-b;4′,3′-e]pyridine (5d): 1H NMR (300.13 MHz, DMSO-d6) δ = 2.07 (s,6H, CH3), 4.78 (s, 1H, CH), 7.04 (d, J = 8.3 Hz, 2H, H-Ar), 7.37 (d, J = 8.4 Hz, 2H, H-Ar), 11.32 (br, s, 3H, NH); 13C NMR (75.47 MHz, DMSO-d6): δ = 10.2,32.2, 104.4, 118.4, 129.7, 130.4, 131.9, 142.7, 157.5.

4-(4-Chlorophenyl)-3,5-dimethyl-1,4,7,8-tetrahydrodipyrazolo[3,4-b:4′,3′-e]pyridine (5e):1H NMR (300.13 MHz, DMSO-d6): δ = 2.06 (s, 6H, CH3), 4.80 (s, 1H, CH),7.12 (d, J = 7.1 Hz, 2H, H-Ar), 7.24 (d, J = 7.2 Hz, 2H, H-Ar), 11.35 (br s, 3H, NH); 13C NMR (75.47 MHz, DMSO-d6): δ = 10.7,32.6, 104.3, 128.0, 129.8, 130.4, 140.1, 142.7, 161.4. IR (KBr, ῡ/cm − 1): 3365, 3100, 2854, 1612, 1526, and 1368.

4-(4-methyl-phenyl)- 3, 5-dimethyl-1, 4, 7, 8-tetrahydro-dipyrazolo [3,4-b:4′,3′-e] pyridine (5f.): 1H NMR (300.13 MHz, DMSO-d6): δ = 2.04 (s, 6H, CH3), 2.21 (s, 3H, CH3), 4.74 (s, 1H, CH), 6.98–7.00 (m, 4 H, H-Ar), 11.24 (s,3H, NH);13C NMR (75.47 MHz, DMSO-d6): δ = 10.8, 20.9, 32.8, 104.8, 127.8, 128.8, 134.7, 140.2,140.7, 161.5.

4-(4-nitro-phenyl)-3,5-Dimethyl-1,4,7,8-tetrahydrodipyrazolo[3,4-b:4′,3′-e]pyridine(5 g):1H NMR (300.13 MHz,DMSO-d6): δ = 2.07 (s, 6H, CH3), 4.95 (s, 1H, CH), 7.34–7.36 (d, 2 H, J = 8 Hz,2H, H-Ar), 8.09–8.11 (d, 2 H, J = 8 Hz, 2H, H-Ar), 11.25 (s, 3H, 3 NH); 13CNMR (75.47 MHz, DMSO-d6): δ = 10.75, 33.43, 103.62, 123.46, 129.25, 140.18,146.09, 152.24, 161.34.

4-(3,5-Dimethyl-1,4,7,8-tetrahydro-dipyrazolo[3,4-b;4′,3′-e]pyridin-4-yl)-phenyl]-dimethylamine(5 h): 1H NMR (300.13 MHz, DMSO-d6) δ = 2.05 (s, 6H, CH3), 2.8 (s, 6H, CH3), 4.69 (s,1H, CH), 6.56(d, J = 8.8 Hz,2H, H-Ar), 6.92 (d, J = 8.5 Hz, 2H, H-Ar), 10.91 (br, s,3H, NH); 13C NMR (75.47 MHz, DMSO-d6):δ = 10.3, 31.7, 40.4, 104.7, 112.2, 127.8, 131.1, 148.5, 159.7, 161.0.

4-(4-Cyano-phenyl)-3, 5-Dimethyl-1, 4, 7, 8-tetrahydrodipyrazolo [3,4-b;4′,3′-e]pyridine(5i):1H NMR (300.13 MHz,DMSO-d6): δ = 2.09 (s, 6H, CH3), 4.93 (s, 1H, CH), 7.32 (d,J = 8.1 Hz, 2H, H-Ar), 7.70 (d, J = 8.1 Hz, 2H, H-Ar), 11.39 (br s,3H, NH); 13C NMR (75.47 MHz, DMSO-d6): δ = 10.77, 33.49, 103.76, 108.72, 119.61, 129.08, 132.19, 140.26,149.91, 161.36.

4-(4-Fluoro-phenyl)-3, 5-Dimethyl-1, 4, 7, 8-tetrahydrodipyrazolo [3,4-b;4',3'e]pyridine(5j):1HNMR(300.13 MHz,DMSOd6): δ = 2.08 (s, 6H, CH3), 4.83 (s, 1H, CH), 7.03 (t, J = 8.9 Hz, 2H, H-Ar),7.15 (dd, J1 = 5.9 Hz, J2 = 8.4 Hz, 2H, H-Ar), 11.35 (br s, 3H, NH)ppm; 13C NMR (75.47 MHz, DMSO-d6): δ = 10.79, 32.57,104.61, 114.74 (J = 21 Hz), 129.67 (J = 7.9 Hz), 139.81(J = 2.8 Hz), 140.10, 160.88 (J = 240 Hz), 161.44.

4-(4-hydroxy-phenyl)-3, 5-Dimethyl-1, 4, 7, 8-tetrahydrodipyrazolo [3,4-b;4′,3′-e]pyridine(5 k):1H NMR (300.13 MHz, DMSO-d6): δ = 2.03 (s, 6H, CH3), 4.65 (s, 1H, CH), 6.56–6.58 (d, J = 8 Hz, 2 H, H-Ar),6.88–6.90 (d, J = 8 Hz, 2H, H-Ar), 9.10 (s, OH), 11.50 (s, 3H, NH); 13CNMR (75.47 MHz, DMSO-d6): δ = 10.32, 31.75, 104.5, 114.42, 128.23, 133.35, 139.76,155.03, 161.04.

4-(4-Methoxy-phenyl)-3, 5-dimethyl-1, 4, 7, 8-tetrahydro-dipyrazolo [3,4-b;4′,3′-e] pyridine (5 l): 1H NMR (300.13 MHz, DMSO-d6): δ = 2.03 (s, 6H, CH3), 3.82 (s ,3H, CH3), 4.53 (s, 1H, CH),7.03 (d, J = 8.7 Hz, 2H, H-Ar), 7.79 (d, J = 8.6 Hz, 2H, H-Ar), 8.99 (br, s, 3H, NH); 13C NMR (75.47 MHz, DMSO-d6): δ = 10.3, 31.9, 54.9, 104.4, 113.0, 128.3, 135.1, 139.6, 157.1, 161.0.

#### The FT-IR spectrum results are characterized in the supplementary information (Figs. S1, S6)

### Antibacterial test

The antibacterial activity of Fe_3_O_4_@Se was tested against five bacterial strains using agar diffusion and colony counting methods. The cultivation perimeter was prepared by dissolving a certain amount of powdered Muller–Hinton agar in distilled water. The Prepared dissolved culture medium and all instruments were sterilized for approximately 15 min at 121 °C and pH = 7.3 in an autoclave.

#### Evaluation of agar diffusion

In disk diffusion tests, the suspensions with 0.5 McFarland turbidity of *S. aureus*, *S.* *saprophyticus*, *P*. *aeruginosa, E. coli,* and *K.* *pneumonia,* strains have been prepared. Then, 10 mg of Fe_3_O_4_@Se nanocomposite powder was added to the Muller-Hinton agar plate containing bacteria, and the sample-loaded disks for the plates were incubated at 37 °C for 24 h. The ability of the Fe_3_O_4_@Se nanocomposite to effectively inactivate bacteria was much greater than that of the other studied samples, including, the bare Fe_3_O_4_ nanoparticles (Figs. 6, S8)^48^.

#### Plate-count method

One of the most important and practical methods for determining the concentration of microbes in a sample is to dilute the sample, grow the microbes on the plates and count the colonies.

*Staphylococcus aureus* and *E. coli* colonies after cultivation for 24 h on Fe_3_O_4_@Se and control samples were compared. As shown in Fig. 7,^48^ the number of bacterial colonies was considerably reduced by treatment with the Fe_3_O_4_@Se nanocomposite in comparison with the control samples of *S. aureus* and *E. coli.*

## Results and discussion

### EDX analysis

**Figure 1:**
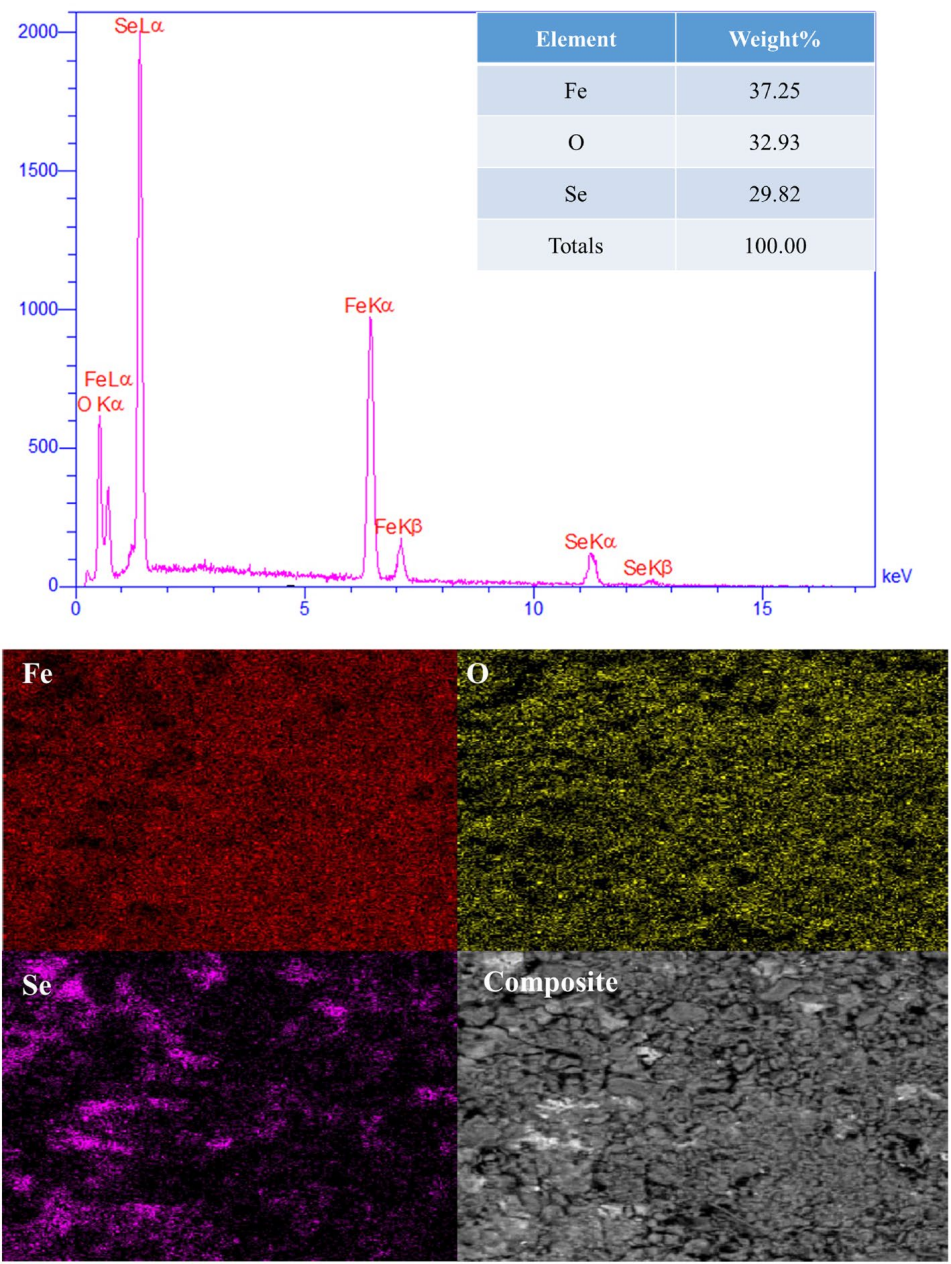
EDS spectra and elemental mapping images of Fe, Se, O, and the Fe_3_O_4_/Se NPs.

EDX was used for the initial analysis of the Fe_3_O_4_/Se nanocomposites. The presence of high-intensity peaks related to O, Fe, and Se indicates nanocomposite components. This is shown in (Fig. 1)^49^. According to the weight percentage table of the components of the Fe_3_O_4_/Se nanocomposite, the most abundant element in this nanocomposite was Fe, which had the highest weight percentage (37.25%), followed by oxygen (32.93%), which had the highest weight ratio, and selenium (29.82), which had the appropriate weight percentage.

### XRD analysis

**Figure 2:**
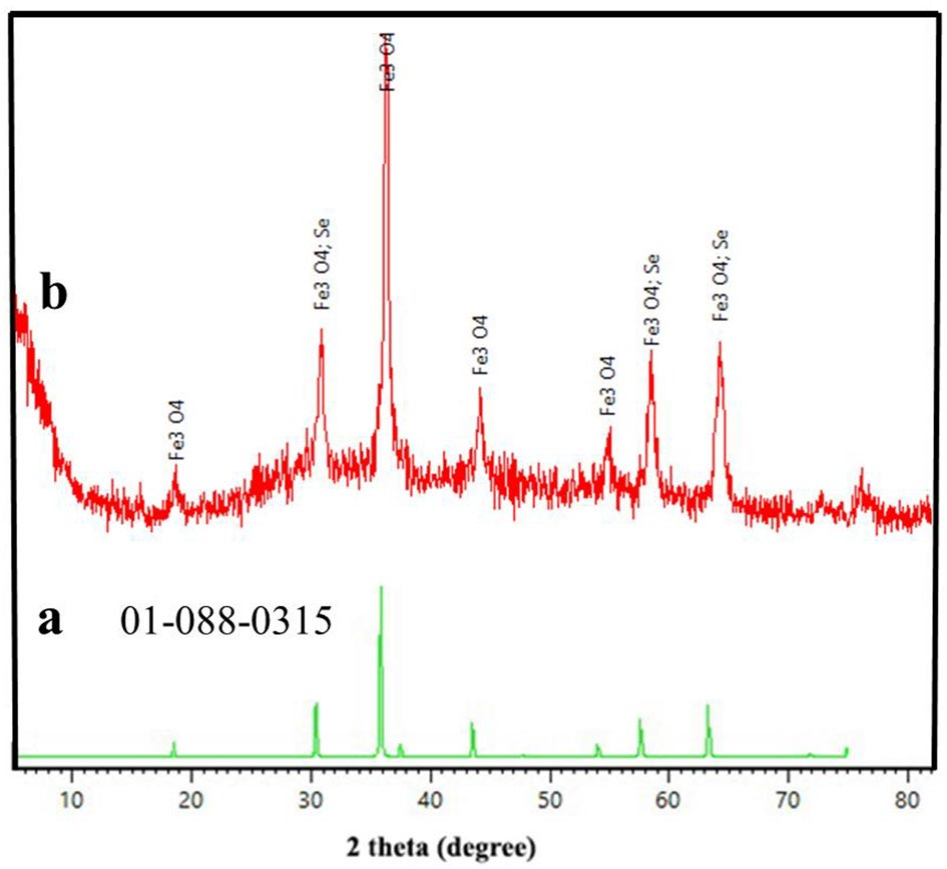
XRD pattern of (**a**) standard Fe_3_O_4_, and (**b**) the Fe_3_O_4_/Se.

The X-ray diffraction (XRD) pattern of the produced Fe_3_O_4_/Se nanocomposite was examined to verify the impact of the composited components on the overall crystal structure (Fig. 2). The diffraction peaks at 2θ = 18.36, 30.2, 35.5, 43.1, 57.1, 62.7, and 74.3 corresponded to the (111), (220), (3 1 1), (400), (4 2 2), (511), (4 4 0), and (533) diffraction planes of magnetite Fe_3_O_4_ NPs with a crystal structure that is in excellent agreement with the reported JCPDS 01-088-0315^37^. Three prominent diffraction peaks at 2θ = 30.141°, 56.999°, and 62.667° are related to the (1 0 1), (1 1 2), and (022) diffraction planes of Se with a crystal hexagonal structure, which is in reasonable agreement with the reported JCPDS card no 01-086-2244. The XRD results for the Fe_3_O_4_/Se NPs indicated that the crystal arrangement of the Fe_3_O_4_ core did not change throughout the functionalization route. Based on this information, the average crystallite size of the Fe_3_O_4_/Se NPs was approximately 20 nm. The crystallite size was calculated by the Debye–Scherrer aquation using Eq. (1), as follows:

1 $$ D_{P} = 0.94\lambda /\beta \cos \theta $$

Since the catalyst has been used several times in the reaction in this regard, the primitive pattern of this nanocomposite was compared with the secondary separate pattern of the Fe_3_O_4_/Se nanocomposite after six cycles of use in the organic reaction pyrazolopyridine, as seen in (Fig. S7), XRD analysis of the consumed catalyst underwent changes after six cycles of use, but there are still index peaks related to the crystal structure of the nanocomposite, which confirms the preservation of the catalyst structure. However, new peaks also appeared, which could be due to impurities in the products or reactants on the surface of the recycled catalyst. (See Supplementary Information).

### FESEM and TEM analysis

**Figure 3:**
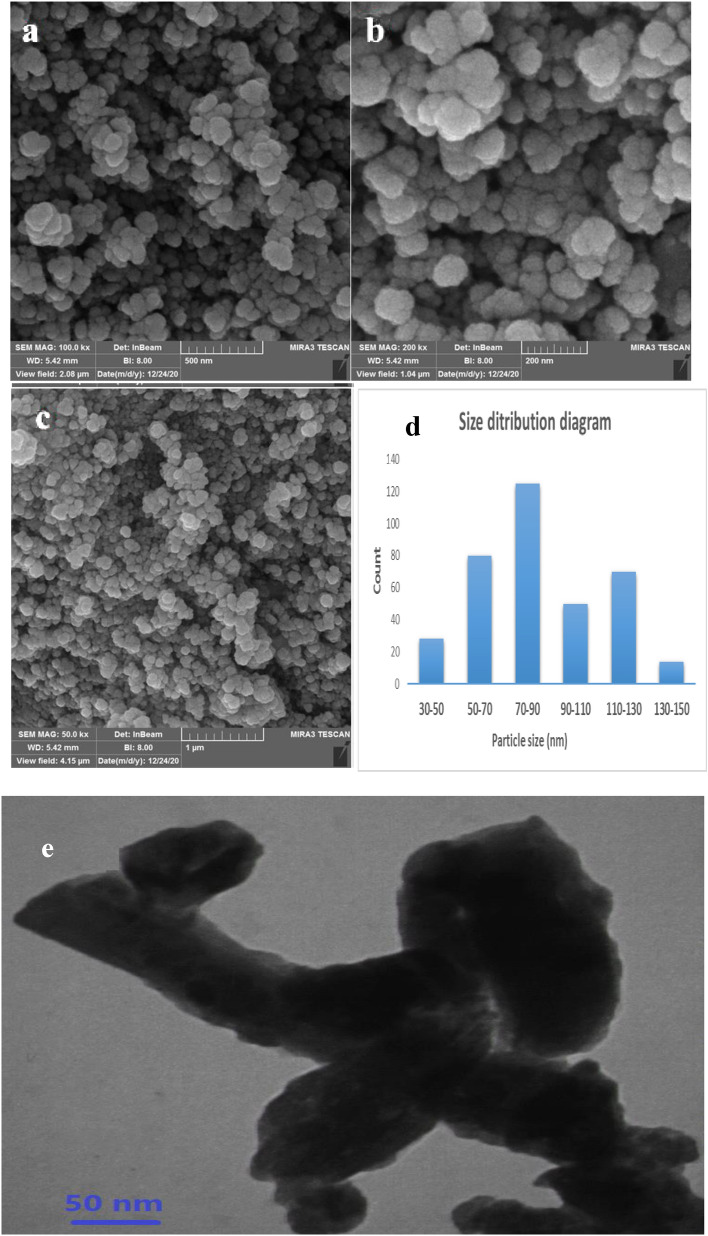
FESEM images of Fe_3_O_4_/SeNPs (**a**) 500 nm (**b**) 200 nm and (**c**) 1 µm and (**d**) particle size distributions (**e**) TEM images of Fe_3_O_4_/SeNPs.

Used to observe particle size distribution, surface morphology, and particle aggregation mode in prepared samples Field-emission scanning-electron microscopy (FESEM) and transmission electron microscopy (TEM) analyses were utilized to examine morphologies and structure. As shown in Fig. 3^4^, the FESEM images of the Fe_3_O_4_/Se NPs are presented at three scales: 1 µm, 500 nm, and 200 nm. The Fe_3_O_4_/Se images showed a spherical structure, in the nanocomposite images, in addition to the spherical structure, the distribution of Se NPs on the Fe_3_O_4_ support was also observed. The average particle size of the 35 spherical particles in the nanocomposite was determined to be approximately 80 nm in addition, As is seen in the TEM image, the spots distributed around the magnetic nanoparticles represent the Se, which confirms the good composition of the Se with the Fe_3_O_4_ also Se acts as the matrix of the inorganic composite.

### The N_2_ adsorption–desorption isotherm

**Figure 4:**
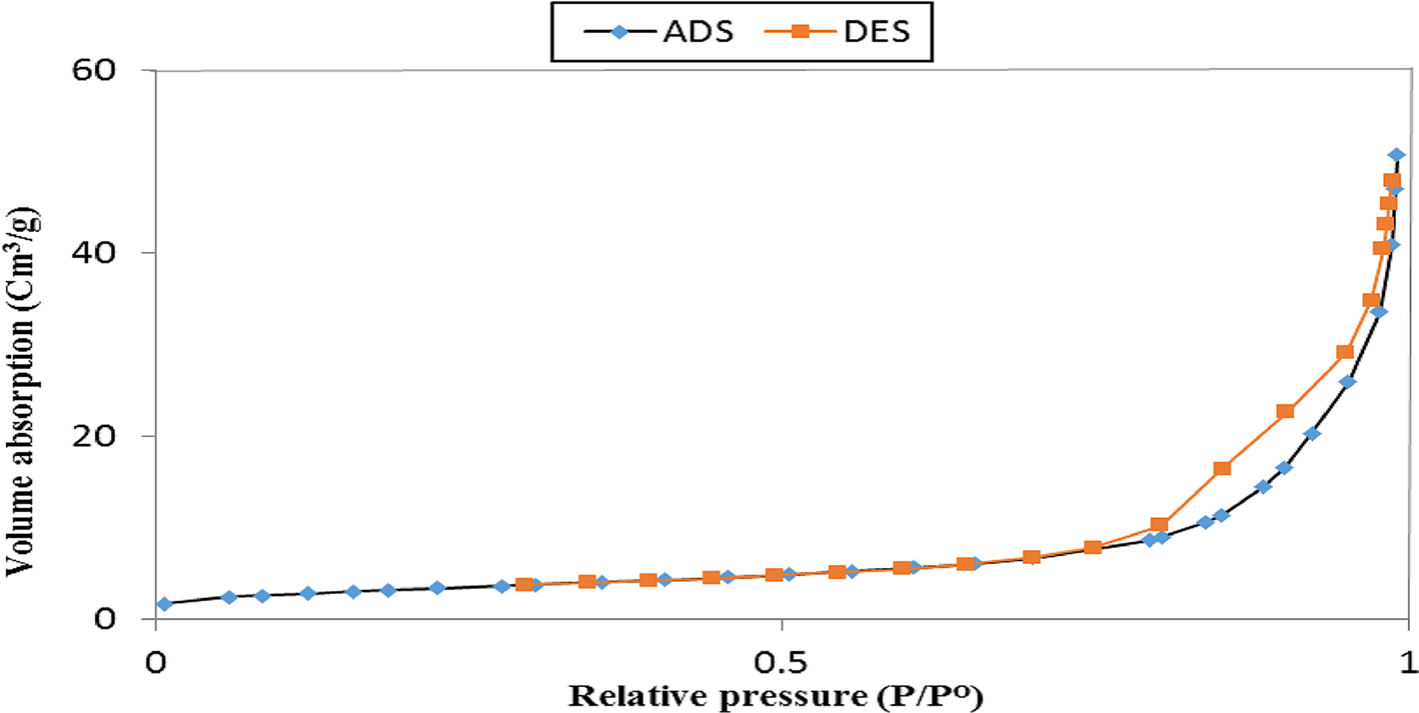
N_2_ adsorption–desorption isotherms of Fe_3_O_4_/Se.

The N_2_ adsorption–desorption isotherm of Fe_3_O_4_/Se is shown in Fig. 4. Detailed information including the surface area, pore volume, and pore size (width) of the Fe_3_O_4_/Se catalytic system, calculated using the (Brunauer–Emmett–Teller (BET)) and (Barrett–Joyner–Halenda(BJH)) methods is presented in Table 1. The BET surface area and pore volume for Fe_3_O_4_/Se were recorded to be approximately 11.57 m^2^/g and 0.073 cm^3^/g, respectively, which are slightly less than reported values in the literature^66,67^ for neat mesoporous Fe_3_O_4_ (15.63 m^2^/g and 0.25960 cm^3^/g). This observation can be mainly related to covering a certain number of Fe_3_O_4_ pores by embedding Se NPs onto the surface of the Fe_3_O_4_ pores, which resulted in less available surface area for gas adsorption and decreased pore volume. However, the Fe_3_O_4_/Se nanocomposite has a high surface area for promoting the catalytic reaction and antibacterial activity.

### VSM analysis

**Figure 5:**
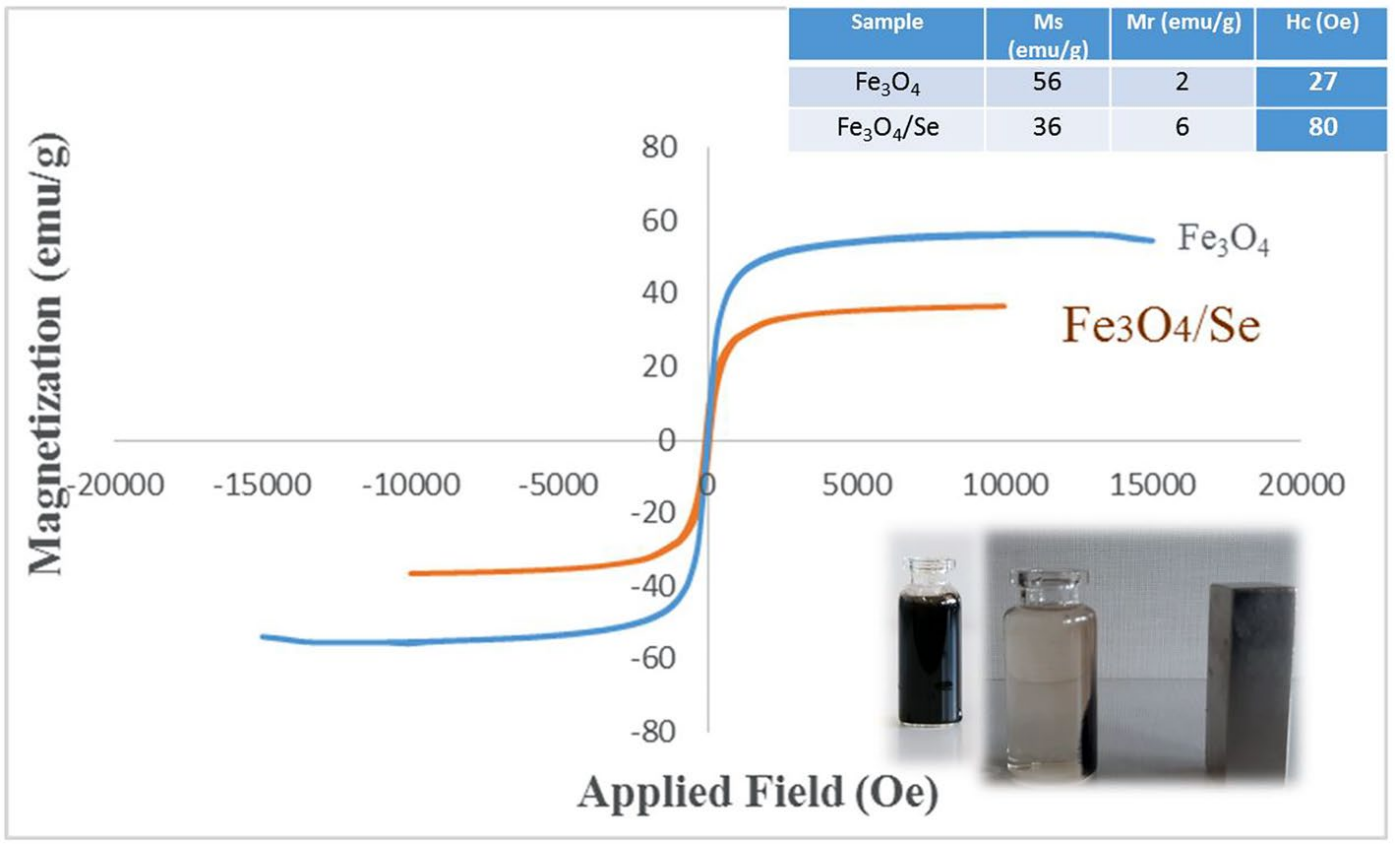
VSM magnetization curve of the Fe_3_O_4_/Se.

**Figure 6:**
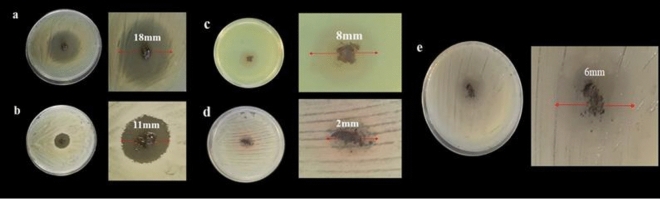
Agar disk diffusions of (**a**) *S. aureus*, (**b**) *E. coli*, (**c**) *P. aeruginosa*, (**d**) *S. saprophyticus,* and (**e**) *K. pneumonia* in the presence of Fe_3_O_4_/Se.

**Figure 7:**
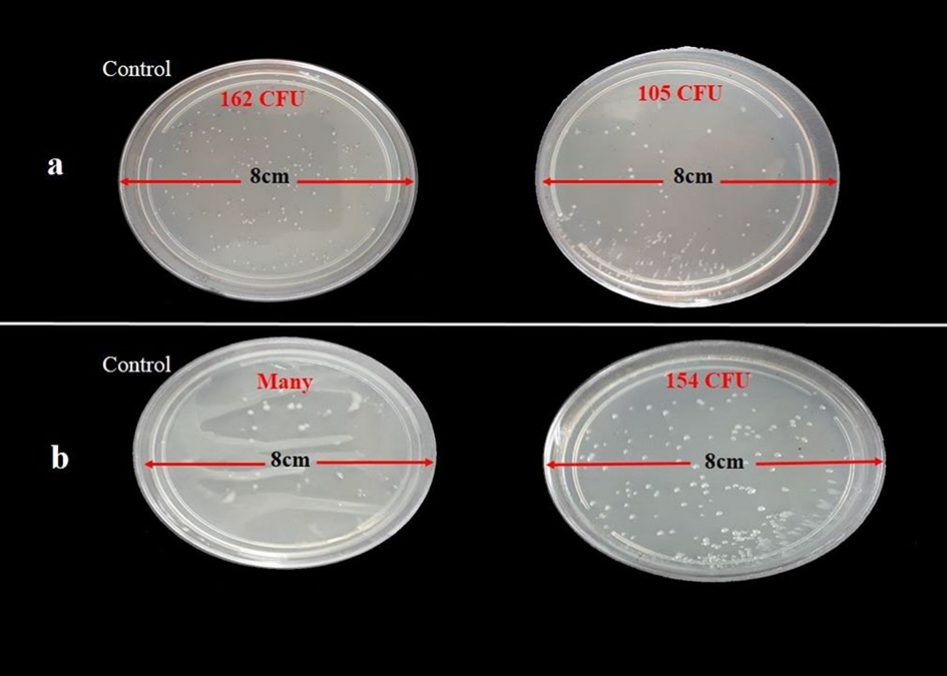
Image colony formation unit (CFU) counting of (**a**) *S. aureus* and (**b**) *E. coli* in the absence and presence (on the right) of Fe_3_O_4_/Se.

VSM analysis was performed to determine the magnetic characteristics of the Fe_3_O_4_/Se hybrid catalyst. As shown in Fig. 5, The S-like magnetization curve showed no hysteresis loop, which indicates that both the remanence (Mr) and coercivity (Hc) were zero and confirmed the superparamagnetic nature of this hybrid catalyst. Magnetic measurements of ambient temperature from − 20,000 to + 20,000 showed that the saturation magnetization (Ms) value of the Fe_3_O_4_/Se nanocomposite compared to that of Fe_3_O_4_ was lowered to 20 emu g^−1^, which was attributed to the addition of selenium to the catalyst reducing the mass percentage of magnetic Fe_3_O_4_/Se. Although Ms decreased, the hybrid catalyst still exhibited strong magnetic activity. And could be collected without difficulty from the reaction mixture using an external magnet (Figs. 6, 7).

### Antibacterial activity

It was discovered that adding Se NPs to the mix might efficiently boost the generation of reactive oxygen species (ROS)^20^. The most widely accepted mechanism of accomplishment for Se NPs is particle attachment to the bacterial surface and the release of selenium ions into the bacterial cell, which results in oxidative stress, protein synthesis inhibition, or DNA mutation^68^.

There are several putative mechanisms of action of Se NPs. Four possible modes of action were examined to determine whether they explain the antibacterial properties of Se NPs: (1) metabolic intrusion by disturbance of intercellular adenosine triphosphate (ATP) concentrations, (2) regulation of the intracellular concentration of reactive oxygen species (ROS), (3) bacterial membrane depolarization, and (4) bacterial membrane disruption. All living organisms use adenosine triphosphate (ATP) as an internal energy source. It is the most important energy source for many enzymatic processes; therefore, it is essential for respiration and metabolism. The energy-uncoupling effect is characterized by the rapid depletion of cellular ATP. Another mechanism that contributes to bacterial mortality is oxidative stress caused by excessive ROS production in response to nanoparticles^69^.

### Catalytic application

Another goal of Fe_3_O_4_/Se nanocatalyst production, as noted in the introduction, is to investigate its catalytic efficacy in organic processes.

In the synthesis of pyrazolopyridine derivatives, the catalytic activity of Fe_3_O_4_/Se was studied.

**Figure 8:**
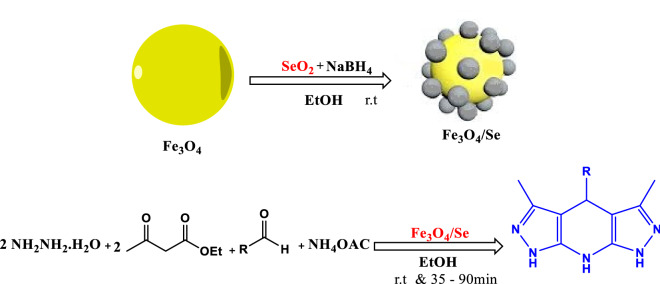
Synthesis of pyrazolopyridine (5a–l) derivatives using Fe_3_O_4_/Se. nanocomposites.

Different experimental conditions such as temperature, solvent, catalyst amount, and catalyst type were investigated in a one-pot four-component reaction of ethyl acetoacetate (2 mmol), hydrazine hydrate (2 mmol), 2, 4-dichlorobenzaldehyde (1 mmol), and ammonium acetate (3 mmol) as a model reaction to obtain the best result. First, the model reaction was carried out at two different temperatures without the use of a catalyst or solvent, and the yield of the products was 37% (Table 2, entry 3). The reaction yield was approximately 37% when the Fe_3_O_4_/Se nanocatalyst was added to the model in the absence of a solvent (Table 2, entry 3). Next, ethanol was added to the reaction in the presence of a catalyst at room temperature to analyze the solvent effect, and the efficiency increased significantly (Table 2, entry 4). The process was then repeated at 80 °C to determine how the temperature affected the outcome, and it was discovered that increasing the temperature to 80 °C caused the reaction to progress (Table 2, entry 5). Subsequently, under reflux and ultrasonic conditions, optimization studies were carried out in H_2_O and EtOH as a green medium; the greatest efficiency was found in EtOH media at room temperature (Table 2, entries 6–8). Various concentrations of nanocatalyst were tested in addition to the reaction conditions and solvent, and the maximum yield of the product was reached in the presence of 0.03 g. (Table 2, entries 9 and 10). In the model reaction, the efficiency of the produced nanocomposite was compared to those of Fe_3_O_4_, Se, and Fe_3_O_4_/Se. The yield of a reaction in the presence of Fe_3_O_4_/Se is higher than Fe_3_O_4_ and Se separately, as indicated (Table 2, entries 12–13). Indeed, an Fe_3_O_4_/Se nanocatalyst with electrophilic selenium nanoparticles, a Lewis acid site (Fe^3+^ in Fe_3_O_4_), and a wide surface area worked as an efficient catalyst for the one-pot four-component reaction to produce pyrazolopyridine derivatives (Fig. 8).

**Table 2:** Optimizing the reaction conditions in the synthesis of pyrazolopyridine **5b**^a^.

Entry	Catalyst	Catalyst loading (g)	Solvent	Temp (°C)	Yield (%)^b^
1	_		_	r.t	Trace
2	_		_	80	Trace
3	Fe_3_O_4_/Se	0.02	_	r.t	37
4	Fe_3_O_4_/Se	0.02	EtOH	r.t	82
5	Fe_3_O_4_/Se	0.02	EtOH	80	87
6	Fe_3_O_4_/Se	0.02	H_2_O	r.t	< 41
7	Fe_3_O_4_/Se	0.02	H_2_O	80	< 50
8	Fe_3_O_4_/Se	0.02	EtOH	Ultrasonic, r.t	84
9	Fe_3_O_4_/Se	0.01	EtOH	80	76
10	Fe_3_O_4_/Se	0.03	EtOH	r.t	96
11	Fe_3_O_4_/Se	0.04	EtOH	r.t	98
12	Fe_3_O_4_	0.03	EtOH	r.t	37
13	Se	0.015	EtOH	r.t	35

^a^Reaction conditions: ethyl acetoacetate (2 mmol), hydrazine hydrate (2 mmol), 3-nitrobezaldehyde (1 mmol) and ammonium acetate (1 mmol), catalyst (0.03 mg).

^b^The yields relate to the isolated product.

#### Examination of the catalytic activity of Fe_3_O_4_/Se

The optimal conditions for synthesizing pyrazolopyridine derivatives using several types of aromatic aldehydes with hydrazine hydrate, ammonium acetate, and ethyl acetoacetate were investigated to assess the generality, application, and limitations between 35 and 90 min at room temperature. All aromatic aldehydes with electron-withdrawing and electron-donating substituents resulted in the synthesis of the corresponding high-yield products, as indicated in Table 3, with no byproducts detected.

**Table 3:** Synthesis of pyrazolopyridine derivatives 5a–l under optimal conditions.

Entry	R	Product	Time (min)	Yield (%)	Mp	
					Found	Reported
1	C_6_H_5_	5a	60	91	241–243	240–242^70^
2	2,4-(Cl)_2_–C_6_H_3_	5b	35	96	310	> 300^62^
3	2-NO_2_–C_6_H_4_	5c	60	93	186–188	187–188^71,72^
4	4-Br–C_6_H_4_	5d	45	95	164–166	165–166^70,72^
5	4-Cl–C_6_H_4_	5e	60	96	254–256	255–257^73^
6	4-Me–C_6_H_4_	5f	75	92	245–247	244–246^74^
7	4-NO_2_–C_6_H_4_	5g	60	93	297–299	295–297^62^
8	4-(Me)_2_N–C_6_H_4_	5h	90	94	240–243	240–242^75^
9	4-CN–C_6_H_4_	5i	40	90	287–289	286–288^62^
10	4-F–C_6_H_4_	5j	90	89	258–260	259–261^62^
11	4-OH–C_6_H_4_	5k	40	97	267–269	269–271^76^
12	4-OMe–C_6_H_4_	5l	80	90	181–183	181–183^76^

#### Proposed mechanism

**Figure 9:**
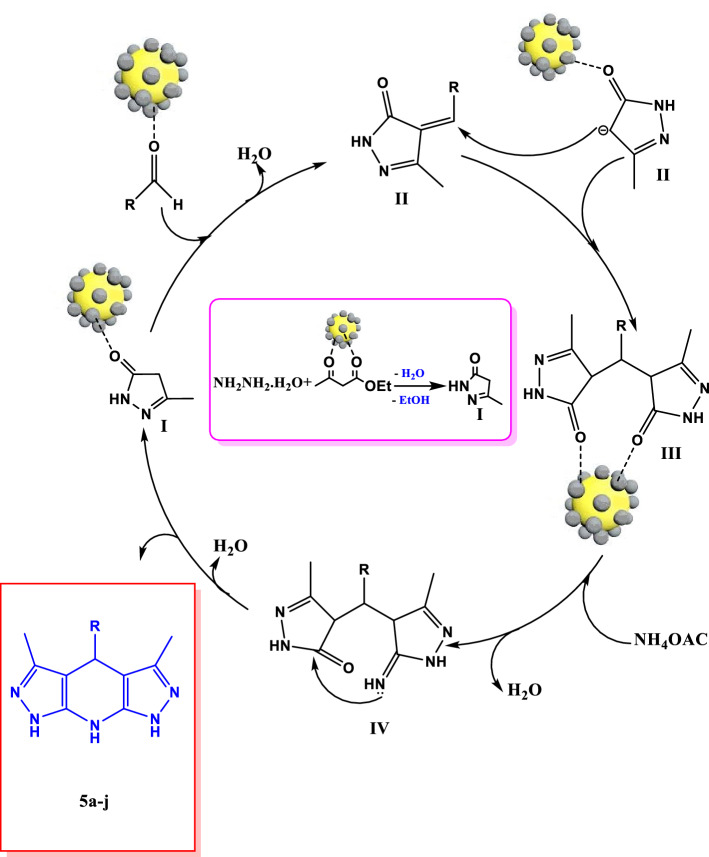
The suggested mechanism for the synthesis of 5a–j by using Fe_3_O_4_/Se.

The most likely mechanism for the synthesis of different pyrazolopyridine derivatives with Fe_3_O_4_/Se nanoparticles is the four-step mechanism shown in Fig. 9. This is not only due to the abundant Lewis acid sites (Fe^3 +^ of Fe_3_O_4_) and, to some extent, the high electrophilic properties of selenium nanoparticles, as well as their physical properties, large surface area, and high thermal and mechanical stability can play an important role in all steps of this four-step reaction illustrated in Fig. 9. Explaining the mechanism of this reaction according to the literature reports^62,70^, first, due to the acidic sites of Fe and the electrophilic property of Se, the oxygen of the carbonyl ethyl acetoacetate groups is involved by the catalyst, which activates the carbonyl groups subjected to the nucleophilic attack of hydrazine hydrate with two nucleophilic sites. In this step, a pyrazolone ring is formed (intermediate I) by removing water and ethanol molecules. Intermediate II was then created by the Knoevenagel condensation of activated aldehydes by the catalyst and pyrazolone ring. The reaction proceeds with the aid of Michael addition by assaulting the second ring of pyrazolone from intermediate (II) and intermediate (III). In the presence of an ammonium acetate catalyst, by attacking intermediate (III), compound IV was formed. In the final stage, compounds 5a-j were synthesized by removing water, performing intramolecular cyclization, and polymerizing compound IV^77,78^. Also, selenium can absorb carbonyl oxygen with its positive charge property.

#### Valuation of the efficiency of Fe_3_O_4_/Se nanocatalyst in the synthesis of pyrazolopyridine derivatives heterocyclic in contrast with other reported studies

A comparison of their catalytic performance was performed to evaluate the benefit of this catalyst over other previously reported catalysts in the synthesis of the 5 k derivative, and the results are shown in Table S1. The results show that the current technology is superior in terms of catalyst biocompatibility, use of an ecologically friendly solvent, and generation of the desired products with high yields in a reasonable amount of time under mild reaction conditions.

### Reusability of Fe_3_O_4_/Se

**Figure 10:**
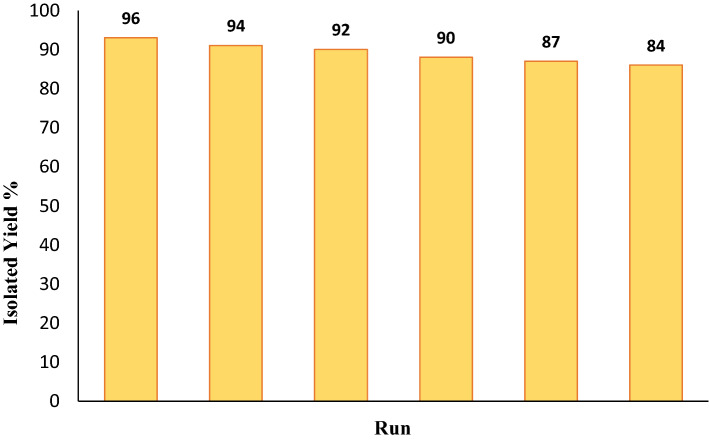
Recyclability of the Fe_3_O_4_/Se catalyst in the synthesis of **5b**.

In terms of industrial and commercial considerations, catalyst reusability is one of the most significant variables, and the ability to recycle products can largely lead the reaction process to conform to the concepts of green chemistry. The recoverability and reusability of the Fe_3_O_4_/Se nanocatalyst were assessed in the synthesis of 5b. Because of the magnetic nature of the catalyst, it can be easily isolated from the reaction mixture using a magnet bar, repeatedly rinsed with distilled water and ethanol, and then dried after each run. Fortunately, after six consecutive cycles, very little catalyst deactivation occurred during pyrazolopyridine synthesis (Fig. 10). The X-ray pattern of the recycled catalyst was almost identical to that of the fresh catalyst (Fig. S7).

## Conclusion

In summary, new, efficient, low-cost, and reusable Fe_3_O_4_/Se NPs were produced and synthesized as nanocomposites using a simple coprecipitation technique. Various analytical methods have been used to evaluate nanocomposite structure, morphology, surface area, pore volume, pore size (width), magnetic properties, and antibacterial characteristics. The SEM images verified the stability and integrity of the spherical structure, as well as the excellent dispersion of Se NPs on the Fe_3_O_4_ surface. The magnetic properties of the nanocomposites were confirmed by VSM analysis. Additionally, detailed information, including the surface area, pore volume, and pore size (width) of the Fe_3_O_4_/Se catalytic system, was calculated using the (Brunauer–Emmett–Teller (BET)) and (Barrett–Joyner–Halenda (BJH)) methods. This shows that the synthesized nanocatalyst had a suitable surface area and pore size to promote organic reactions. The nanocomposite was employed as a well-organized and recyclable heterogeneous nanocatalyst for the production of pyrazolopyridine products via a four-component reaction. At room temperature and under mild reaction conditions, high to excellent product yields were achieved using Fe_3_O_4_/Se as a nanocatalyst. The catalyst can be simply removed from the reaction medium by an external magnetic field, washed and dried, and used several times without a substantial loss of activating sites. The antibacterial properties of this nanocomposite were investigated in the removal and destruction of G^+^ *S. aureus*, *S. saprophyticus*, G^−^ *E. coli*, *K. pneumonia, P. aeruginosa* bacteria, a group of dangerous bacteria that threaten the health of living organisms. This nanocomposite can also be utilized to disinfect water polluted with bacteria; the inactivation of *S*. *aureus* and *E. coli* in the presence of nanoparticles was confirmed by the colony method. Moreover, this is the first report on the plan, production, functionalization, and characterization of the current nanocomposite, as well as its presentation as a heterogeneous nanocatalyst in a significant organic process. Pyrazolopyridine is one of the most important heterocyclic compounds with many biological activities. In this paper, we report an effective and practical method for the synthesis of pyrazolopyridine and its derivatives.

## Supplementary Information

Supplementary Information.

## Data Availability

All data generated or analysed during this study are included in this published article [and its supplementary information files].
