# Supplementary material for: Synthesis and characterization of Se doped Fe3O4 nanoparticles for catalytic and biological properties
Source: Sci Rep. 2023 Jan 18;13:1007. doi: 10.1038/s41598-023-28284-x (PMC9849448; doi:10.1038/s41598-023-28284-x)
Supplement: Supplementary file 1 — Supplementary Information. [file 41598_2023_28284_MOESM1_ESM.doc]

**Synthesis and Characterization of** **Se doped Fe_3_O_4_ nanoparticles and study of their application as heterogeneous catalysts and** **antibacterial properties**

Mohammad Reza Ahghari, Zeinab Amiri-khamakani^1^ and Ali Maleki*

*Catalysts and Organic Synthesis Research Laboratory, Department of Chemistry, Iran University of Science and Technology, Tehran 16846-13114, Iran*

*^1^co-first author*

**Corresponding author.* Fax: +98-21-73021584; Tel: +98-21-73228313;

*E-mail:* [*maleki@iust.ac.ir*](mailto:maleki@iust.ac.ir)

| **Table of contents** |
| --- |
| **Subject Page** |
| **Figure S1.** FT-IR spectra of the of the product(C_6_H_5_) **5a** ……………………..…….…………..……….S2 |
| **Figure S2.** ^1^H NMR spectrum of the product **5a** ……………………..…………….....………………….S3 |
| **Figure S3.** ^13^C NMR spectrum of the product **5a**  …………………...………………………….….……..S4 |
| **Figure S4.** ^1^H NMR spectrum of the product **5e**  ………………………………………………..….……...S5  **Figure S5.** ^13^C spectrum of the product **5e** ………………………………………………………………S6 |
| **Figure S6.** FT-IR spectra of the of the product (4-Cl- C_6_H_4_) **5e**.………..……………………..….….……S7 |
| **Figure S7.** Comparisonthe X-ray diffraction pattern of a. the nanocatalyst after the 6 times reactions b. the nanocatalyst fresh…………………………………………………………………………………………...S8  **TableS1.** Comparison of catalyst the amount and effect with other synthesized catalysts……………….S9  **Figure S8.** Antibacterial effects of Fe_3_O_4_ NPs (a) *S. aureus*, (b) *E. coli*, (c) *P. aeruginosa*, (d) *S.saprophyticus* and (e) *K. pneumonia*……………………………………………………………………S10 |


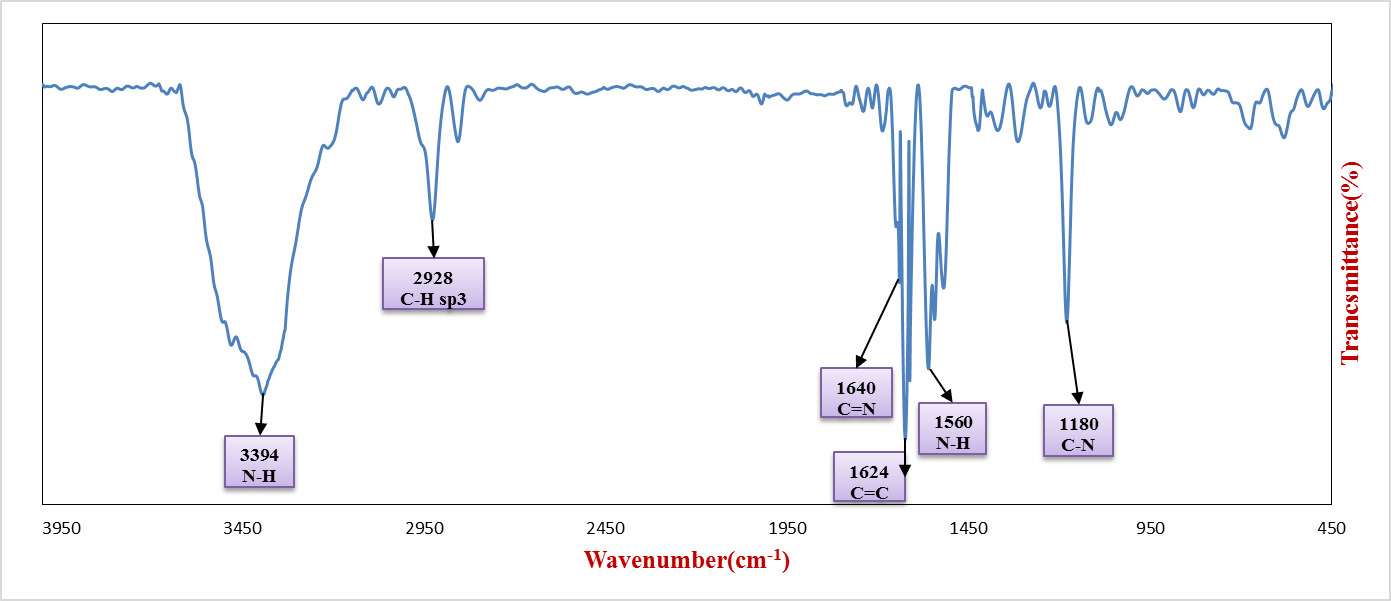


**Figure S1.** FT-IR spectrum of the product **5a**.


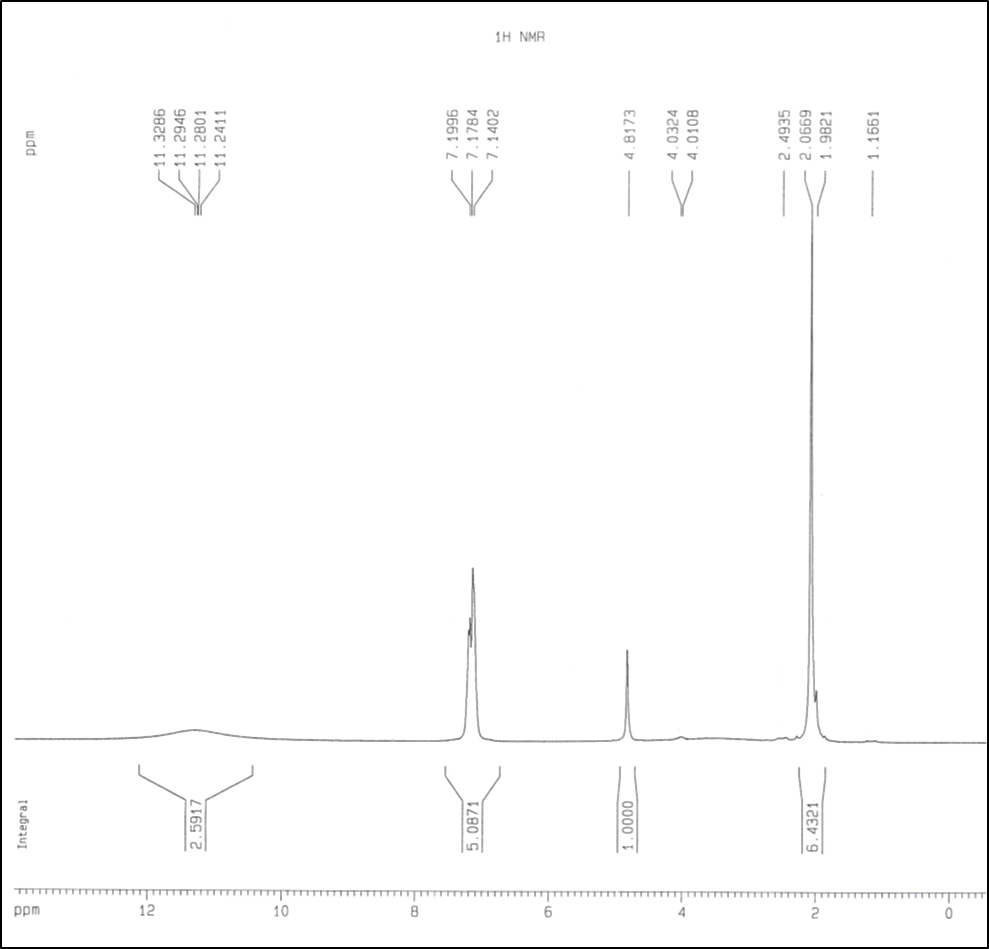


**Figure S2.** ^1^H NMR spectrum of the product **5a.**


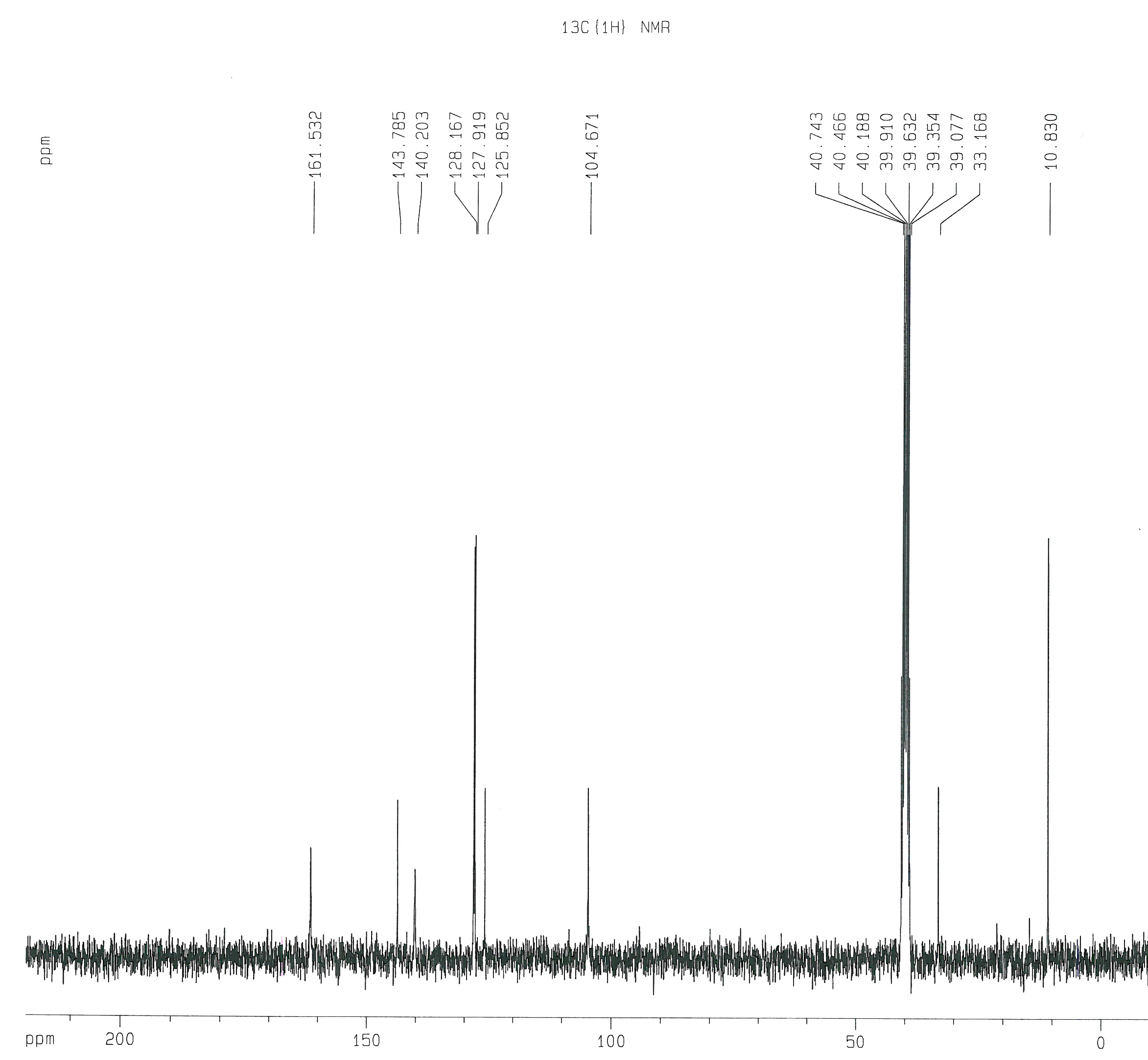


**Figure S3.** ^13^C NMR spectrum of the product **5a**.


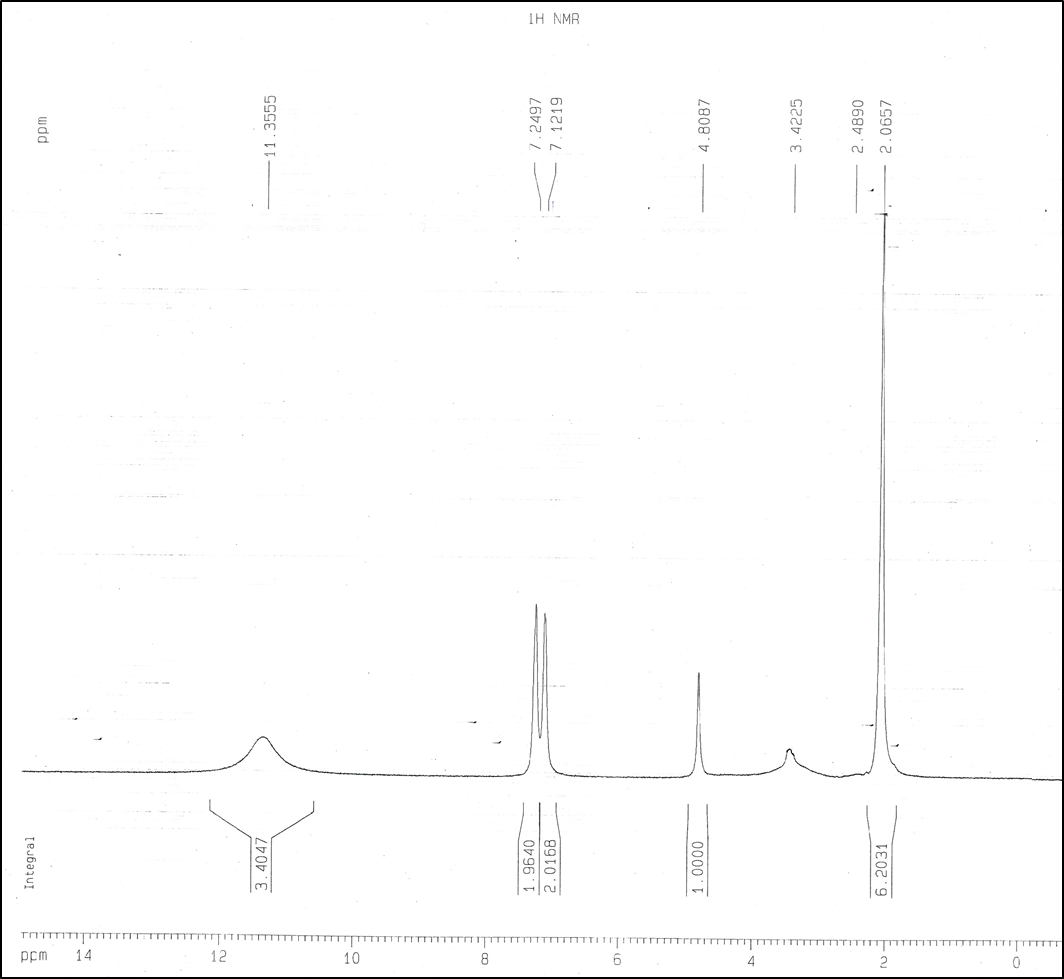


**Figure S4.** ^1^H NMR spectrum of the product **5e.**


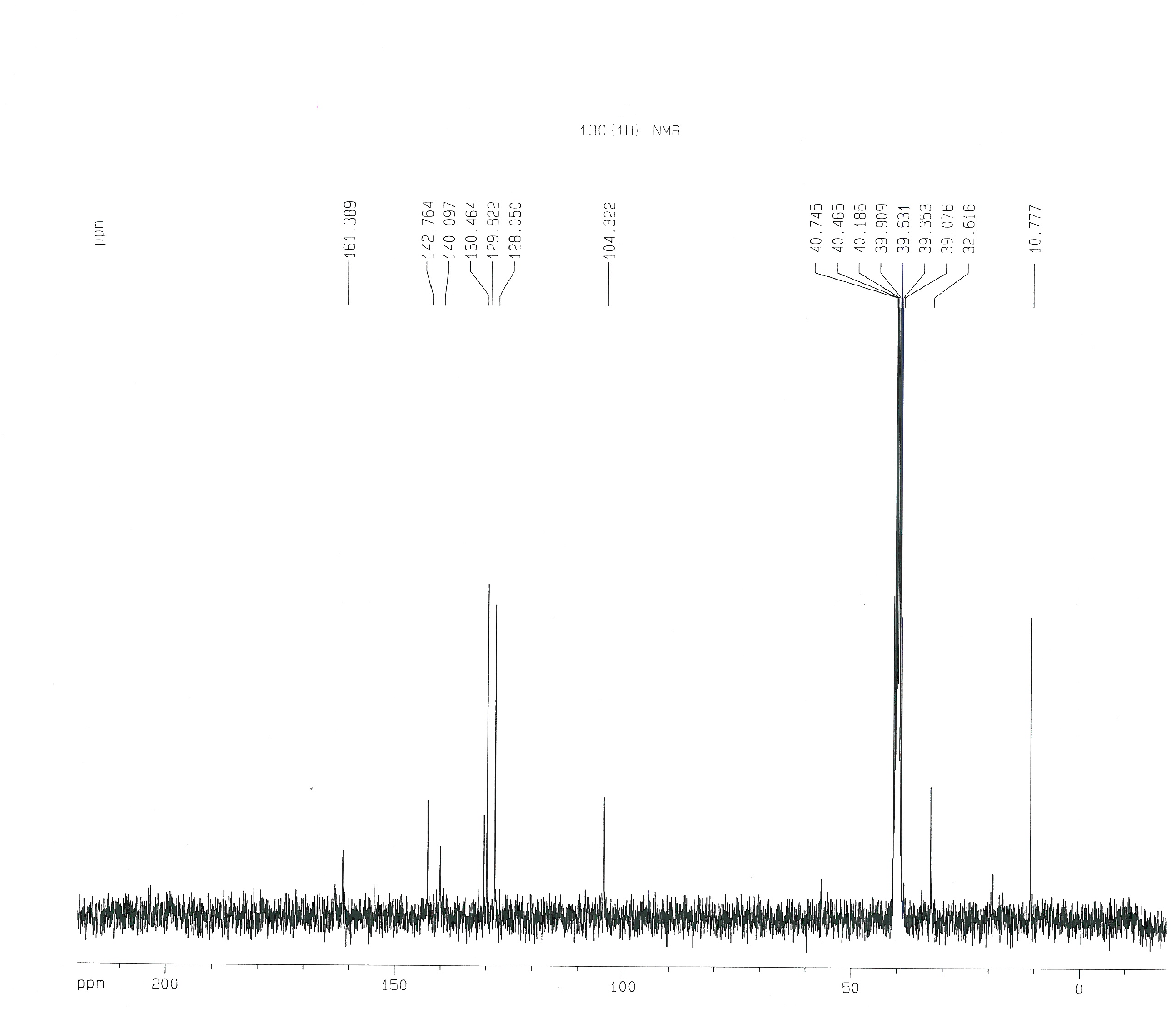


**Figure S5.** ^13^C NMR spectrum of the product **5e**.


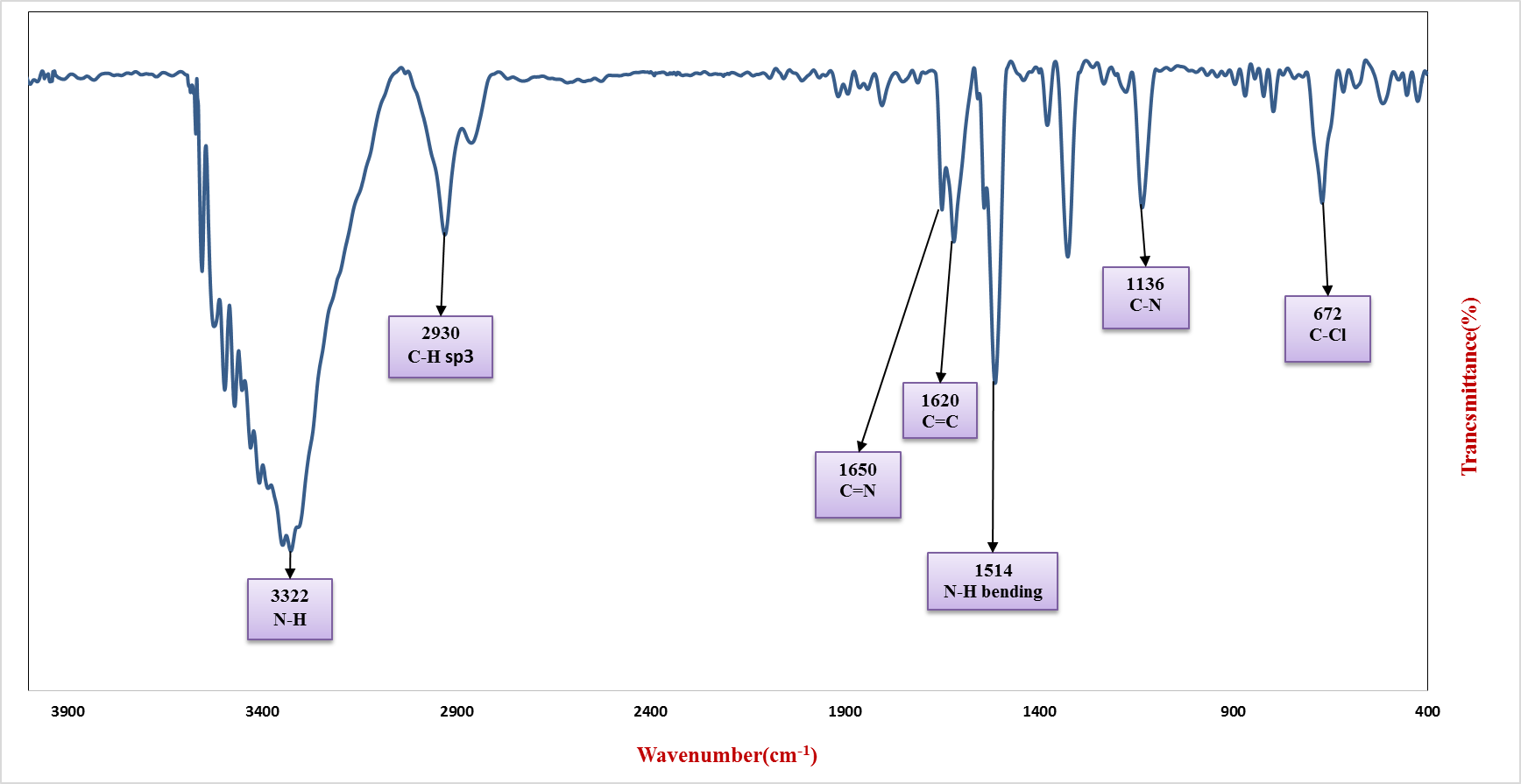


**Figure S6.** FT-IR spectrum of the product **5e**.


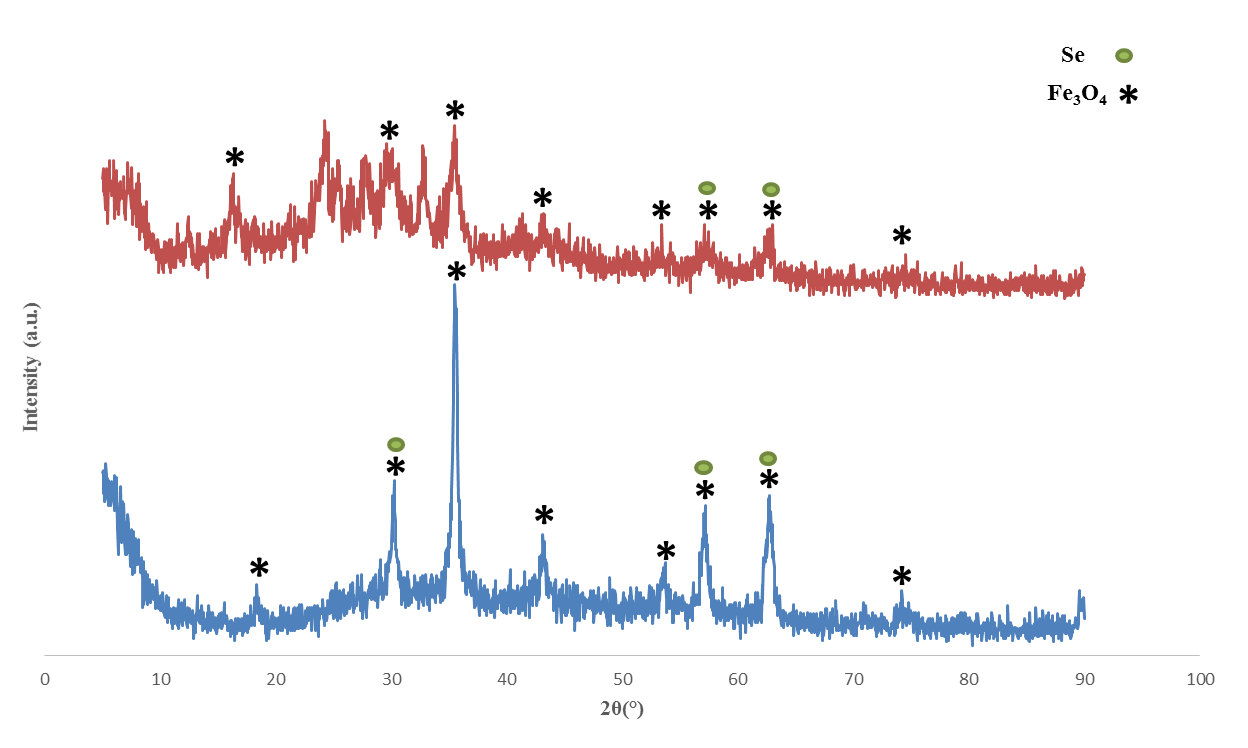


**a**

**b**

**Figure S7.** Comparison the X-ray diffraction pattern of a. the nanocatalyst after the 6 times reactions b. the nanocatalyst fresh[7,8]

| Entry | Catalyst | Catalyst loading | Conditions | Yield^a^ (%) | Ref. |
| --- | --- | --- | --- | --- | --- |
| 1 | ....... | ....... | H_2_O,Ultrasonic, 70 °C | 60 | [1] |
| 2 | HANCD@urease | 0. 1g | H2O, 70 °C | 90 | [2] |
| 3 | nano-CdZr4(PO4)6 | 0.06 mol% | EtOH, reflux | 80 | [3] |
| 4 | Fe3O4/KCC-1/IL/HPW | 0.1g | H2O.r.t | 90 | [4] |
| 5 | CuFe2O4@HNTs | 0.5g | EtOH,r.t | 90 | [5] |
|  | CuCr2O4 NP | 4 mol% | EtOH,r.t | 84 | [6] |
| 6 | **Fe3O4/Se** | **0.03g** | **EtOH,r.t** | **97** | ….. |

**TableS1.** Comparison of catalyst the amount and effect with other synthesized catalysts


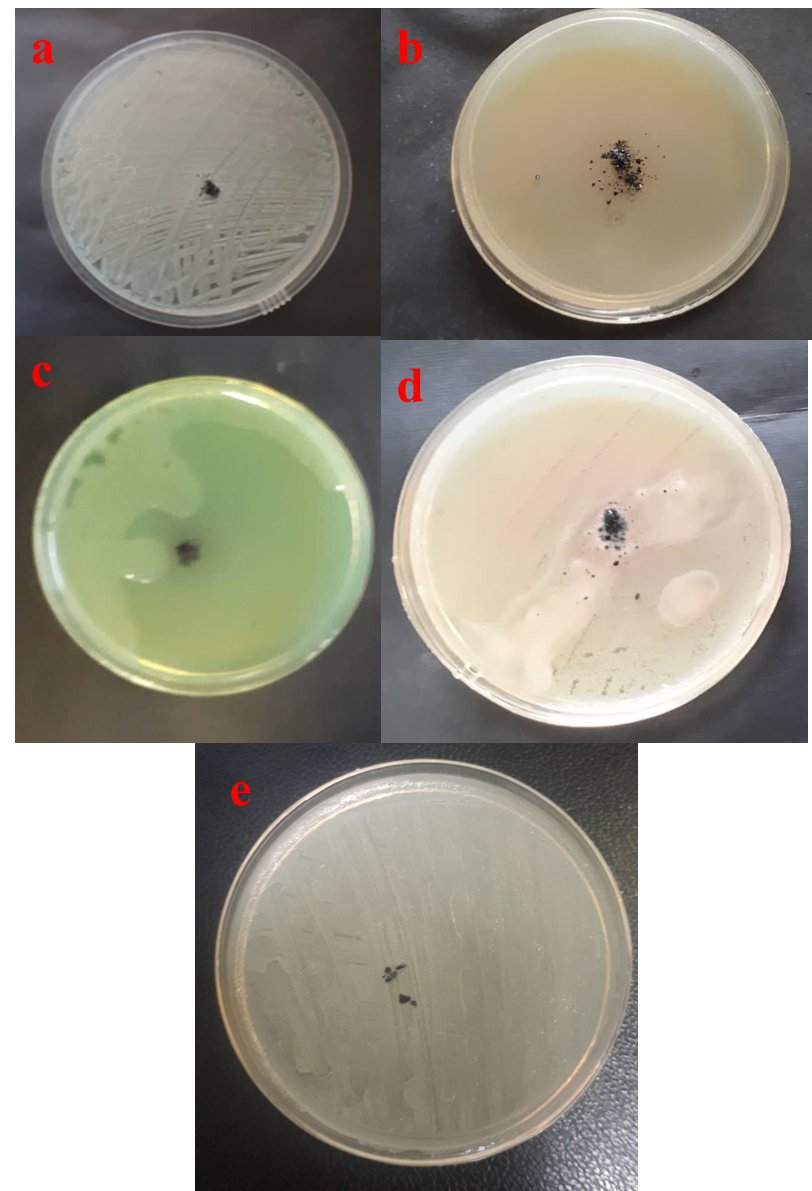


**Figure S8.** Antibacterial effects of Fe_3_O_4_ NPs (a*) S. aureus*, (b) *E. coli*, (c) *P. aeruginosa*, (d) *S. saprophyticus* and (e) *K. pneumonia*[9]

1. Shabalala, Nhlanhla Gracious, Ramakanth Pagadala, and Sreekantha B. Jonnalagadda. "Ultrasonic-accelerated rapid protocol for the improved synthesis of pyrazoles." *Ultrason Sonochem* **27** (2015): 423-429.
2. Tamaddon, Fatemeh, and Davood Arab. "Urease covalently immobilized on cotton-derived nanocellulose-dialdehyde for urea detection and urea-based multicomponent synthesis of tetrahydro-pyrazolopyridines in water." *RSC Adv* **9**, no. 71 (2019): 41893-41902.
3. Safaei-Ghomi, Javad, Hossein Shahbazi-Alavi, Reyhaneh Sadeghzadeh, and Abolfazl Ziarati. "Synthesis of pyrazolopyridines catalyzed by nano-CdZr4 (PO4) 6 as a reusable catalyst." *Research on Chemical Intermediates* 42, no. 12 (2016): 8143-8156.
4. Sadeghzadeh, Seyed Mohsen. "A heteropolyacid-based ionic liquid immobilized onto magnetic fibrous nano-silica as robust and recyclable heterogeneous catalysts for the synthesis of tetrahydrodipyrazolopyridines in water." *RSC Adv* 6, no. 79 (2016): 75973-75980.
5. Maleki, Ali, Zoleikha Hajizadeh, and Peyman Salehi. "Mesoporous halloysite nanotubes modified by CuFe2O4 spinel ferrite nanoparticles and study of its application as a novel and efficient heterogeneous catalyst in the synthesis of pyrazolopyridine derivatives." *Sci. Rep*. 9, no. 1 (2019): 1-8.
6. Shahbazi-Alavi, Hossein, Javad Safaei-Ghomi, Fahime Eshteghal, Safura Zahedi, Seyed Hadi Nazemzadeh, Fatemeh Alemi-Tameh, Maryam Tavazo, Hadi Basharnavaz, and Mohammad Rasool Lashkari. "Nano-CuCr2O4: an efficient catalyst for a one-pot synthesis of tetrahydrodipyrazolopyridine." *Journal of Chemical Research* 40, no. 6 (2016): 361-363.
7. Sasaki, S.A.T.O.S.H.I., 1997. Radial distribution of electron density in magnetite, Fe3O4. *Acta Crystallogr B: Structural Science*, 53(5), pp.762-766.
8. Das, S., Priyadarshini, P., Alagarasan, D., Vardhrajperumal, S., Ganesan, R. and Naik, R., 2022. Structural, morphological, and linear/non-linear optical properties tuning in Ag60-xSe40Tex films by thermal annealing for optoelectronics*. J Non Cryst Solids*, 592, p.121742.
9. Zare-Bakheir, Ensiye, Mohammad Reza Ahghari, Ali Maleki, and Hossein Ghafuri. "Synthesis of Cu (OH) 2 nanowires modified by Fe_3_O_4_@ SiO_2_ nanocomposite via green and innovative method with antibacterial activity and investigation of magnetic behaviours." *R Soc Open Sci* 9, no. 6 (2022): 212025.
